# Supplementary material for: DLAB: deep learning methods for structure-based virtual screening of antibodies
Source: Bioinformatics. 2021 Sep 21;38(2):377–83. doi: 10.1093/bioinformatics/btab660 (PMC8723137; doi:10.1093/bioinformatics/btab660)
Supplement: btab660_Supplementary_Data [file btab660_supplementary_data.zip › supplementary_table_I.PDF]

Antibody name	Non-cognate RBD variant mutations	PDB code of complex	Reference
AB1	E406W<br>N487R<br>K417N, E484K, N501Y	7mjl	Sun *et al.* (2021)
S2X259	G504D	7m7w	Tortorici *et al.* (2021)
BG10-19	K417N, E484K, N501Y	7m6e	Scheid *et al.* (2021)
BG1-22	K417N, E484K, N501Y	7m6f	Scheid *et al.* (2021)
BG4-25	K417N, E484K, N501Y	7m6d	Scheid *et al.* (2021)
S2-M28	L18F, R246I, D80A	7ly0	McCallum *et al.* (2021)
S2-L28	L18F, R246I, D80A, D215G<br>L18F<br>R246G<br>D253G<br>D253Y	7lxx	McCallum *et al.* (2021)
S2-X333	L18F, R246I, D80A	7lxw	McCallum *et al.* (2021)

Supplementary Table 1: Overview of the SARS-CoV2 variant binding dataset. Note that 7mjl was backmutated Y501N to restore wild-type, as no complex structure against the wild-type RBD was available. The triple mutant K417N, E484K, N501Y characterises the variant of concern B.1.351

References

McCallum, M. *et al.* (2021). N-terminal domain antigenic mapping reveals a site of vulnerability for SARS-CoV-2. *Cell*, **184**(9), 2332–2347.e16.

Scheid, J. F. *et al.* (2021). B cell genomics behind cross-neutralization of SARS-CoV-2 variants and SARS-CoV. *Cell*, **184**(12), 3205–3221.e24.

Sun, Z. *et al.* (2021). Neutralization of European, South African, and United States SARS-CoV-2 mutants by a human antibody and antibody domains. *bioRxiv*. 2021.03.22.436481.

Tortorici, M. A. *et al.* (2021). Broad sarbecovirus neutralization by a human monoclonal antibody. *Nature*, **597**(7874), 103–108.
