# Supplementary figures and images for: DLAB: deep learning methods for structure-based virtual screening of antibodies
Source: Bioinformatics. 2021 Sep 21;38(2):377–83. doi: 10.1093/bioinformatics/btab660 (PMC8723137; doi:10.1093/bioinformatics/btab660)

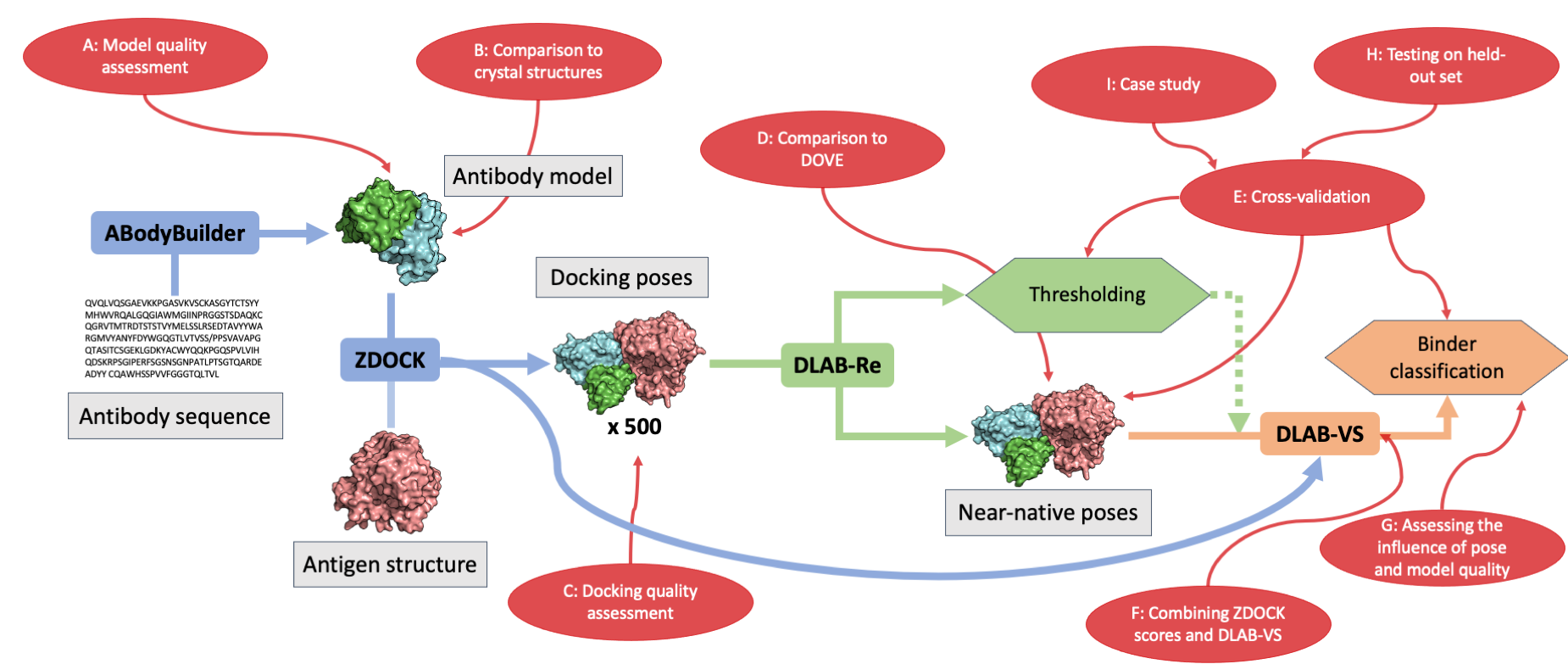

Supplement: btab660_Supplementary_Data [file btab660_supplementary_data.zip › supplementary_figure_1.png]

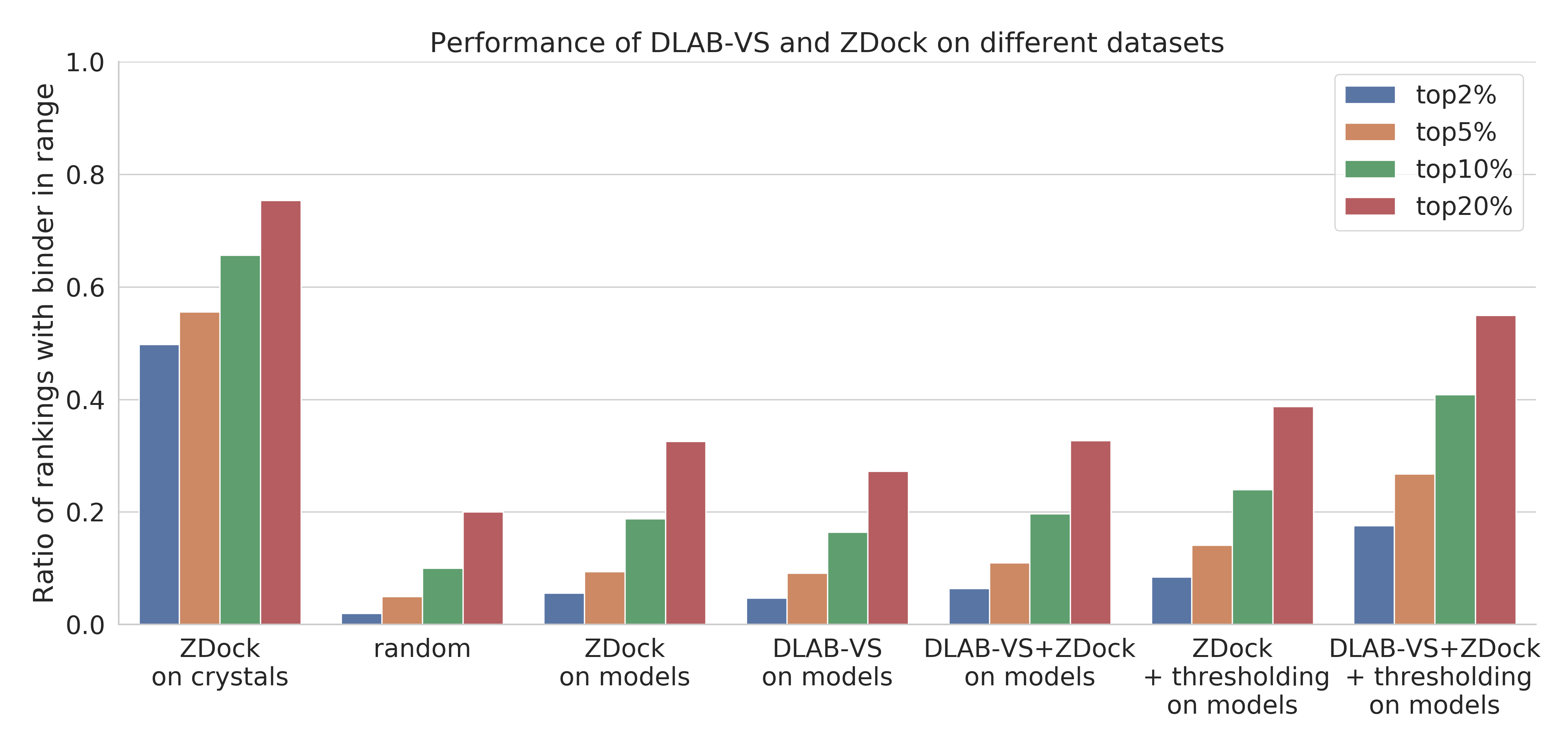

Supplement: btab660_Supplementary_Data [file btab660_supplementary_data.zip › supplementary_figure_10.png]

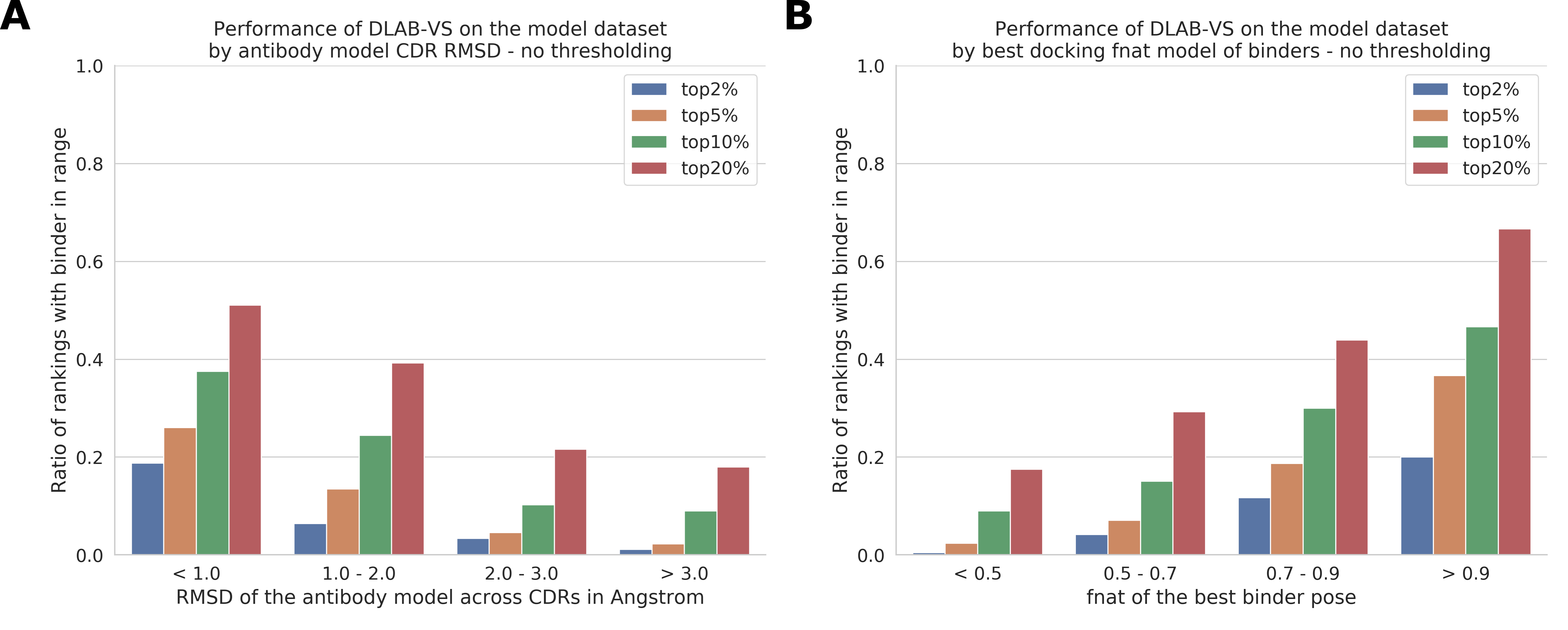

Supplement: btab660_Supplementary_Data [file btab660_supplementary_data.zip › supplementary_figure_11.png]

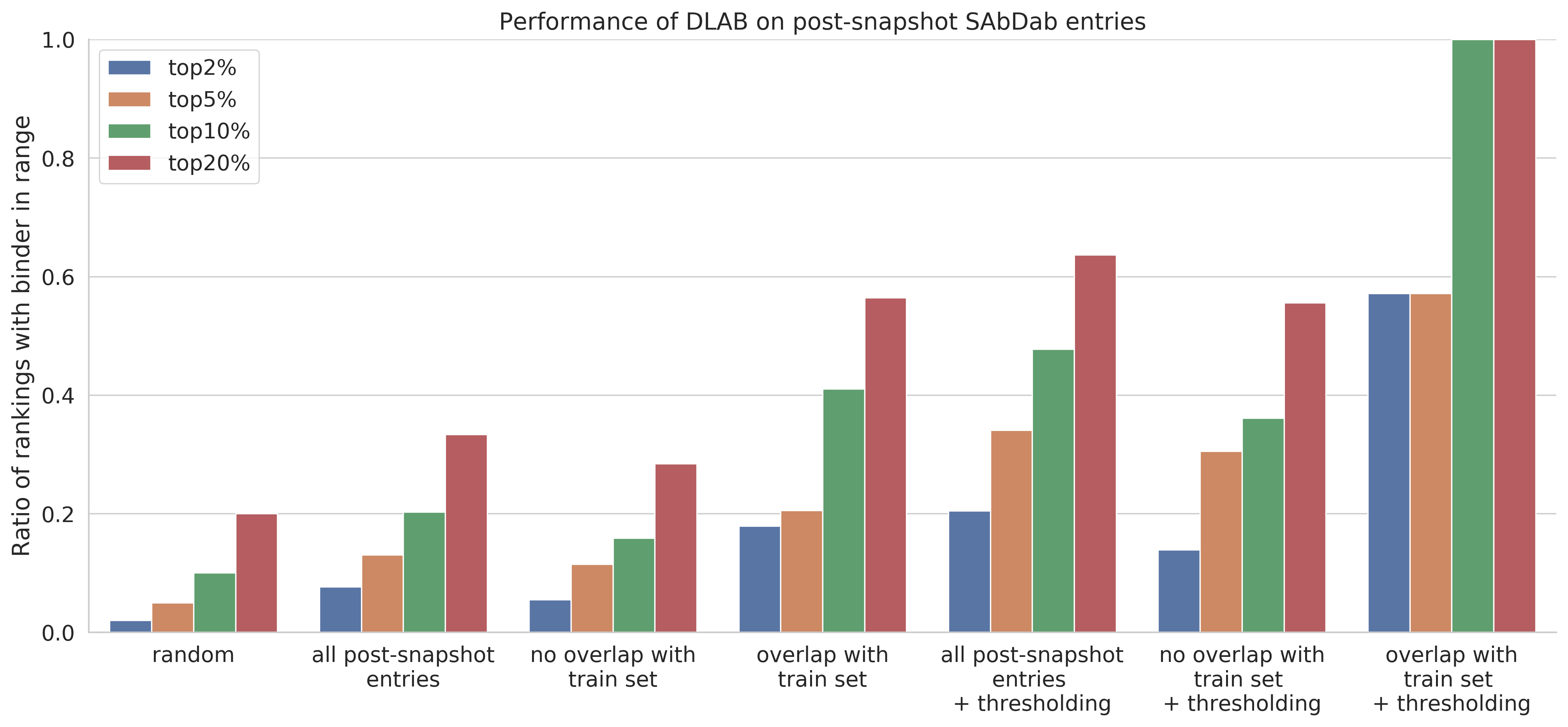

Supplement: btab660_Supplementary_Data [file btab660_supplementary_data.zip › supplementary_figure_12.png]

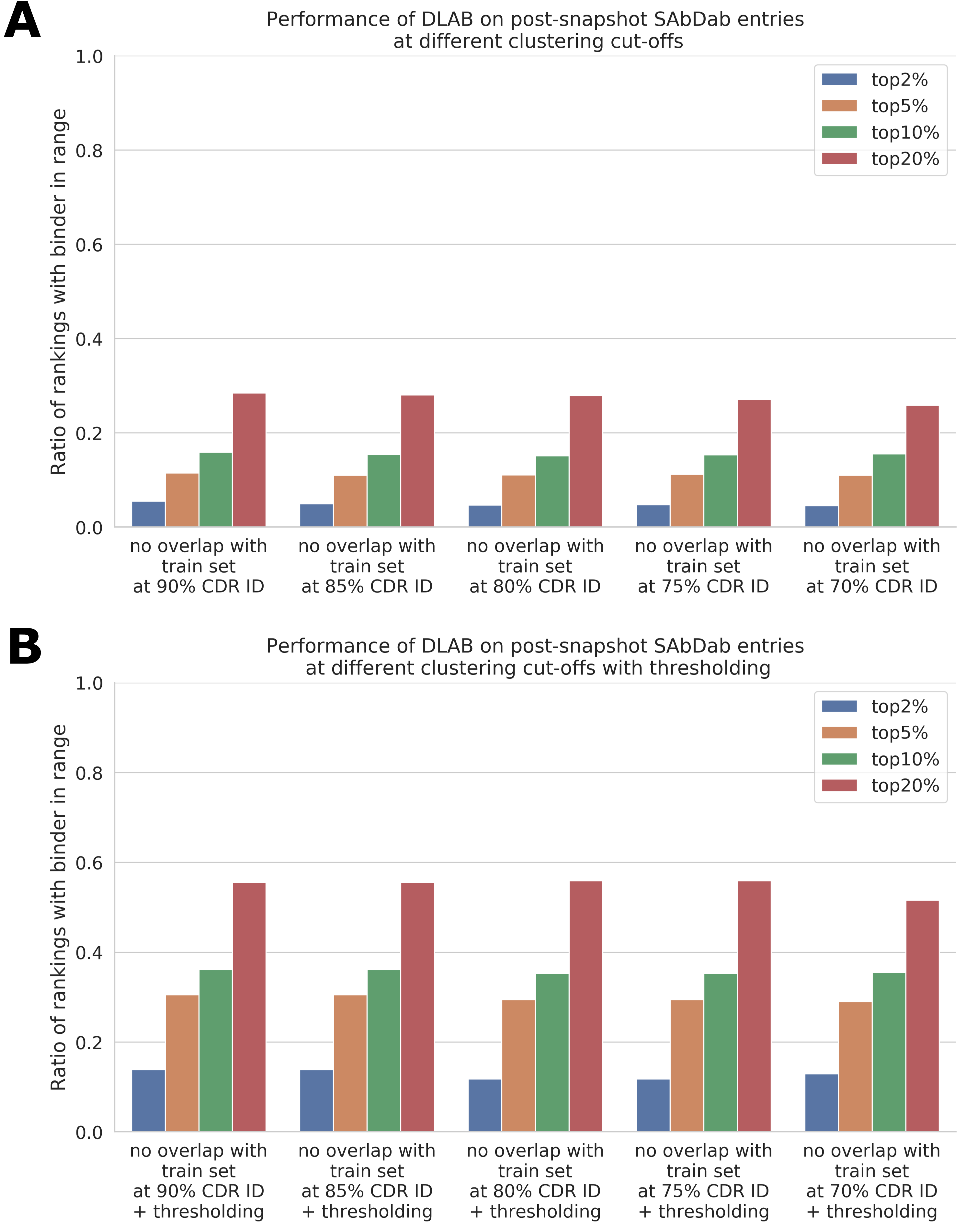

Supplement: btab660_Supplementary_Data [file btab660_supplementary_data.zip › supplementary_figure_13.png]

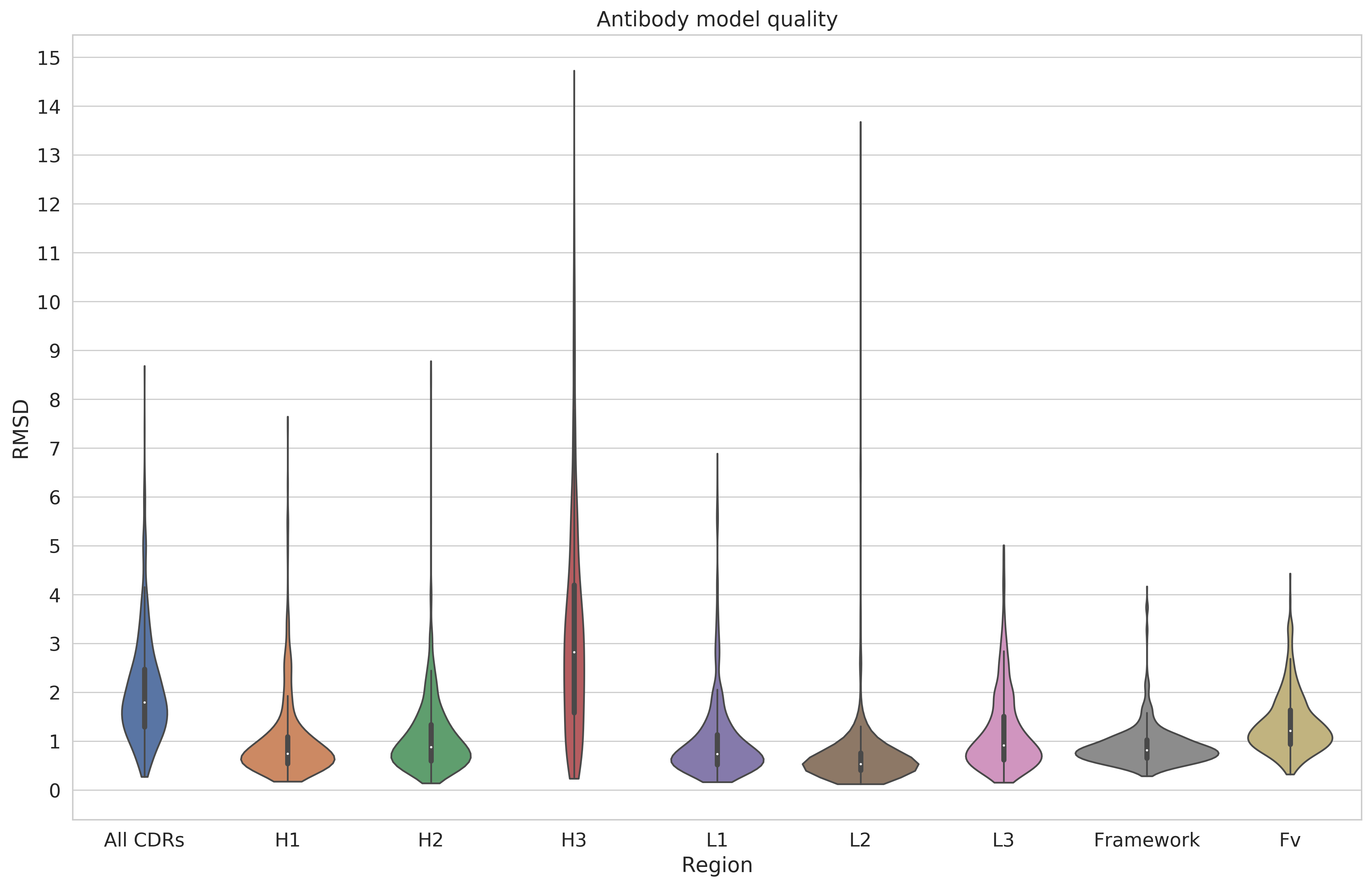

Supplement: btab660_Supplementary_Data [file btab660_supplementary_data.zip › supplementary_figure_2.png]

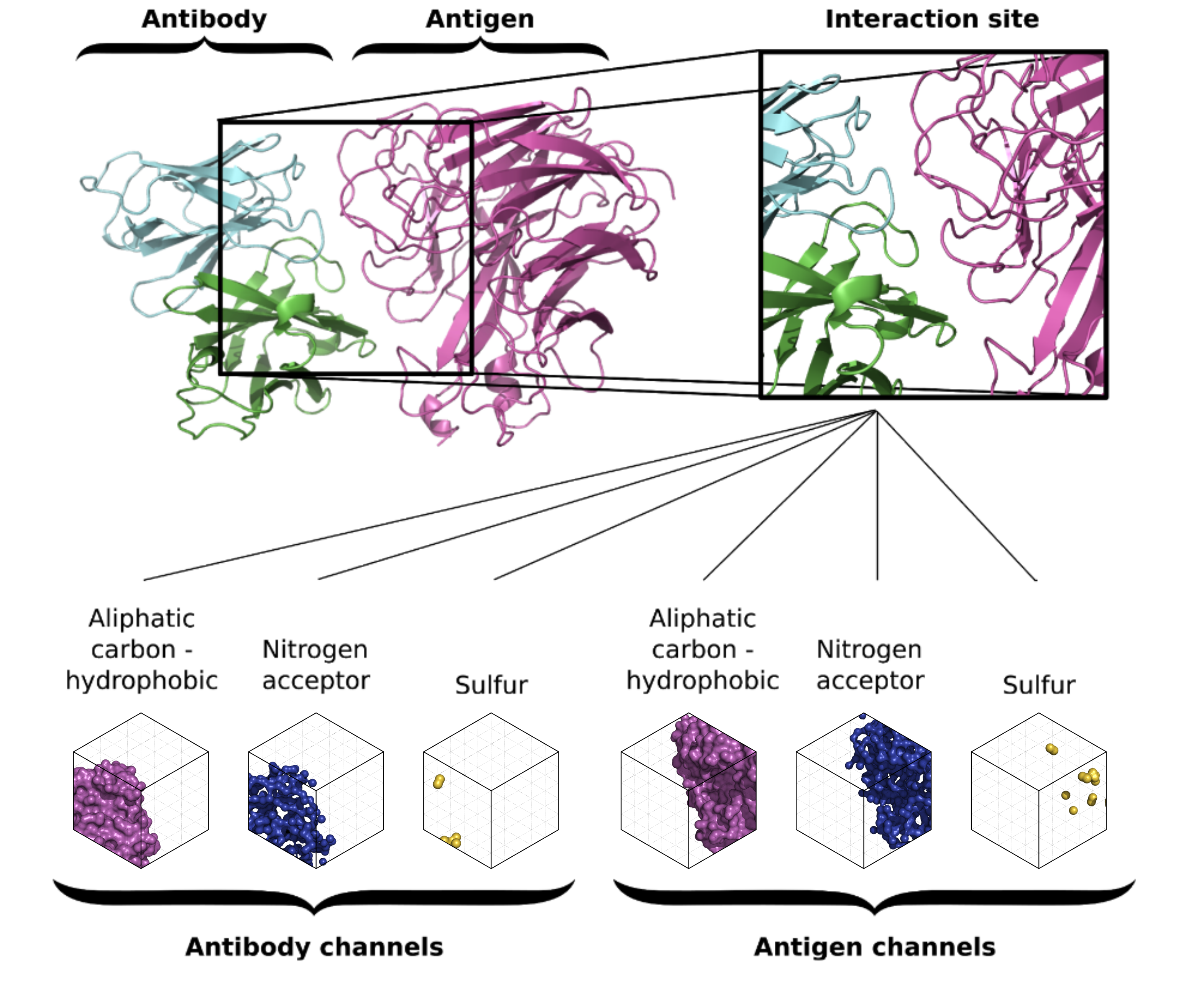

Supplement: btab660_Supplementary_Data [file btab660_supplementary_data.zip › supplementary_figure_3.png]

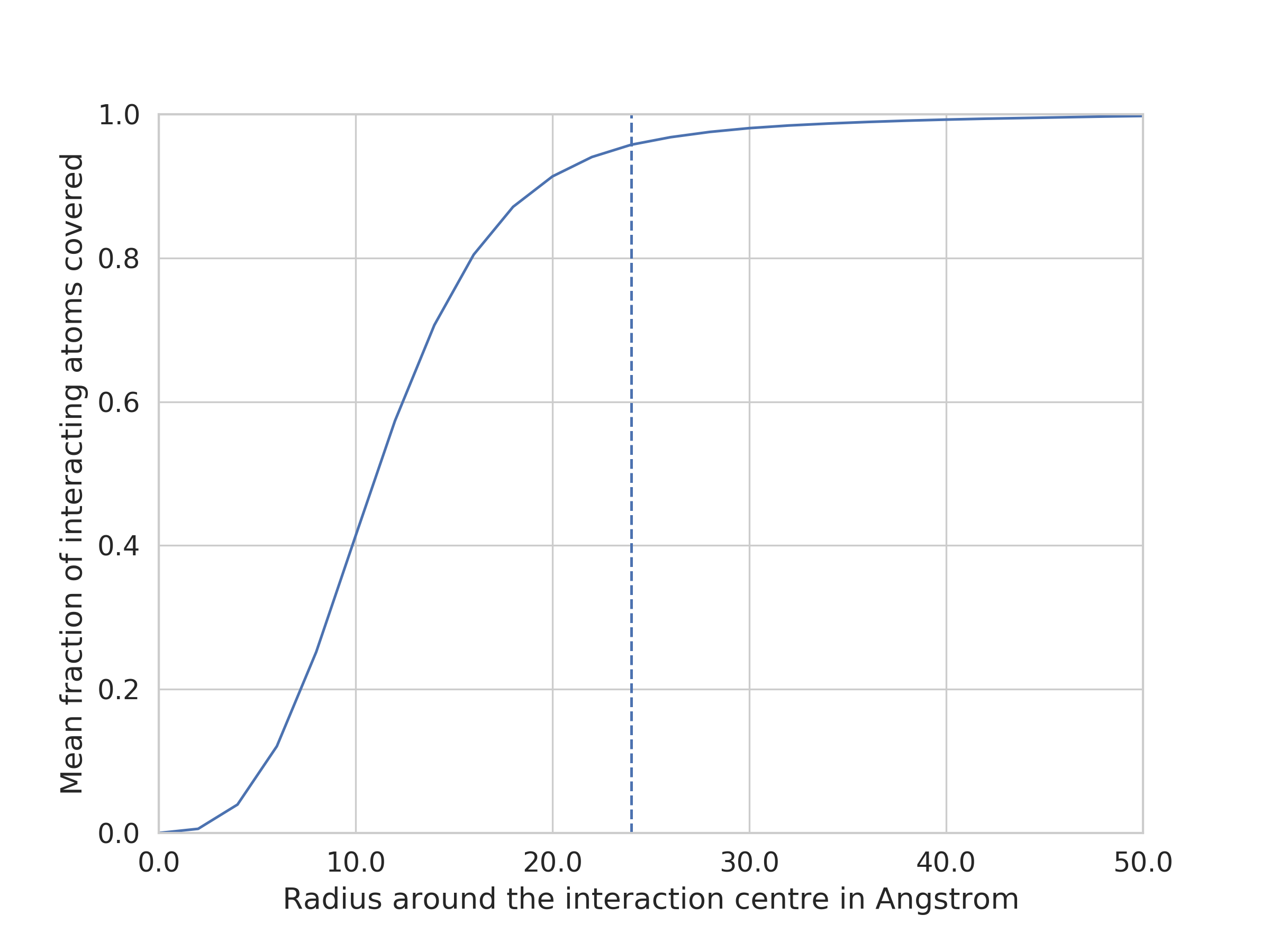

Supplement: btab660_Supplementary_Data [file btab660_supplementary_data.zip › supplementary_figure_4.png]

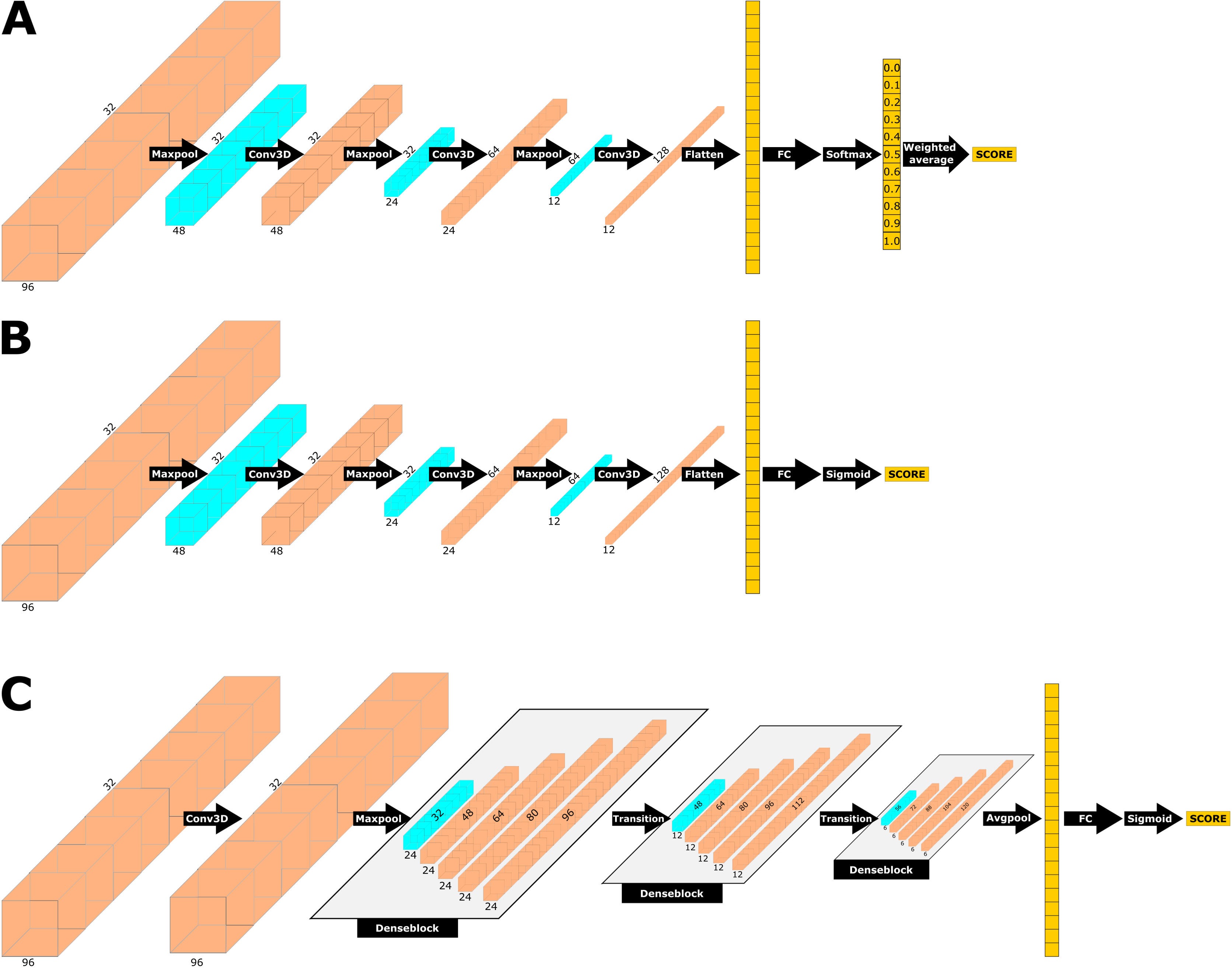

Supplement: btab660_Supplementary_Data [file btab660_supplementary_data.zip › supplementary_figure_5.png]

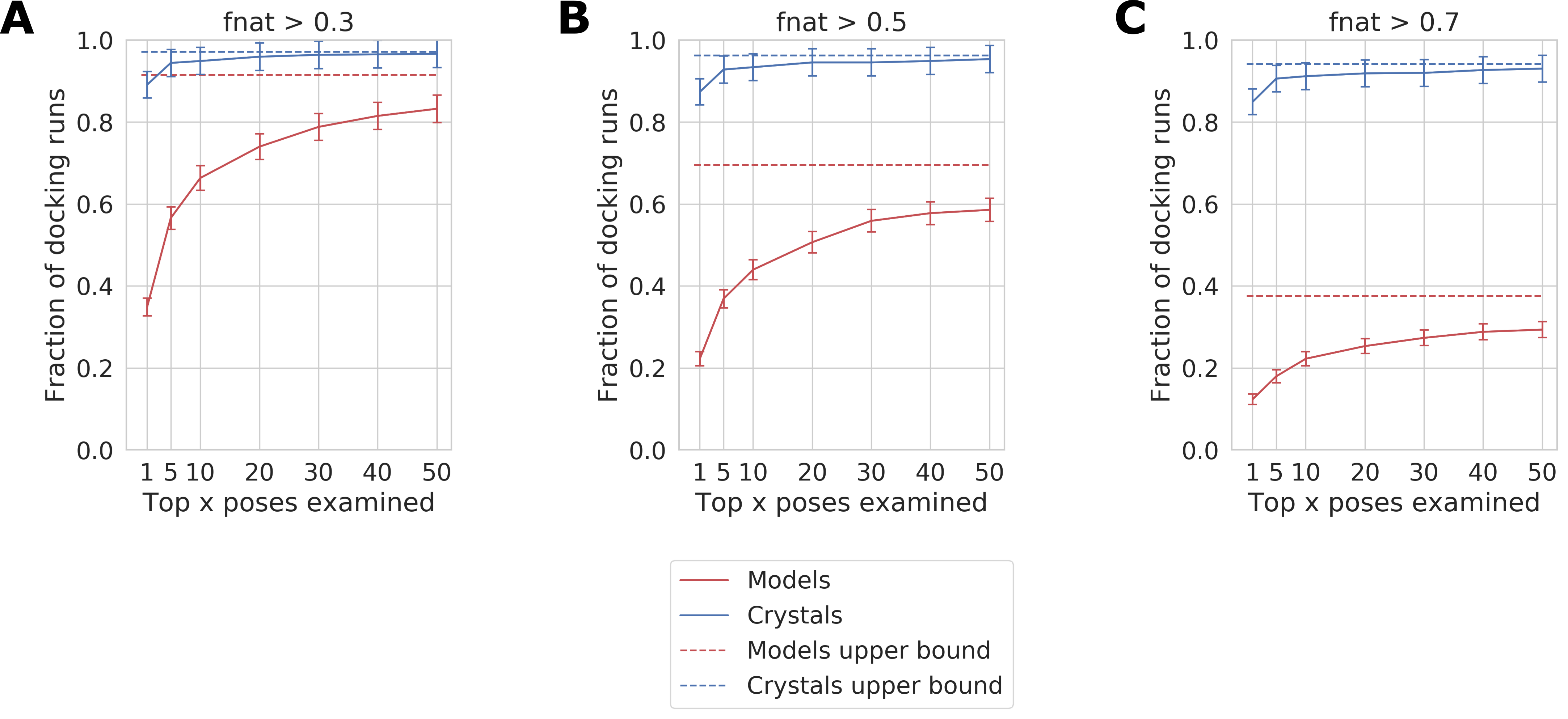

Supplement: btab660_Supplementary_Data [file btab660_supplementary_data.zip › supplementary_figure_6.png]

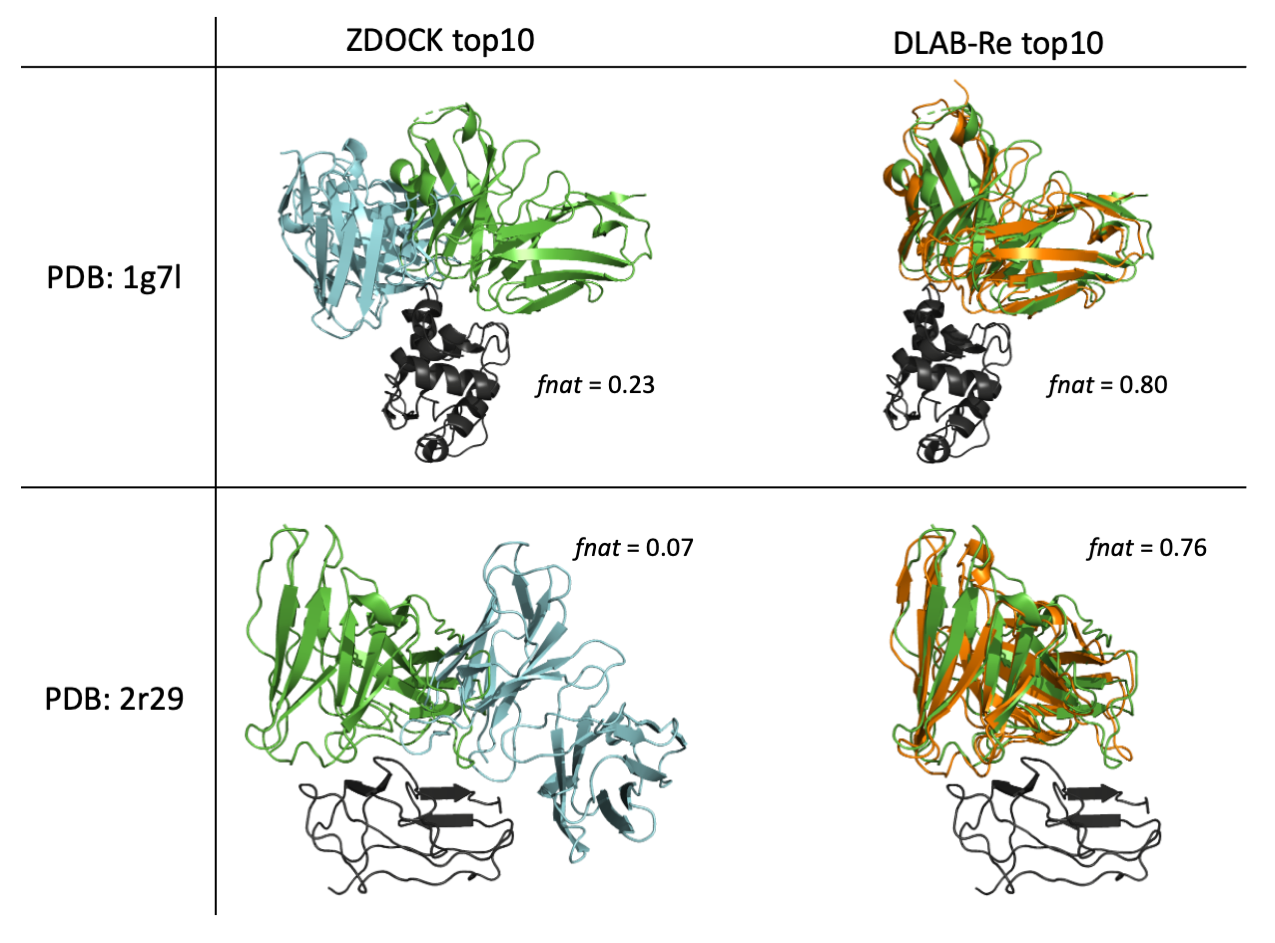

Supplement: btab660_Supplementary_Data [file btab660_supplementary_data.zip › supplementary_figure_7.png]

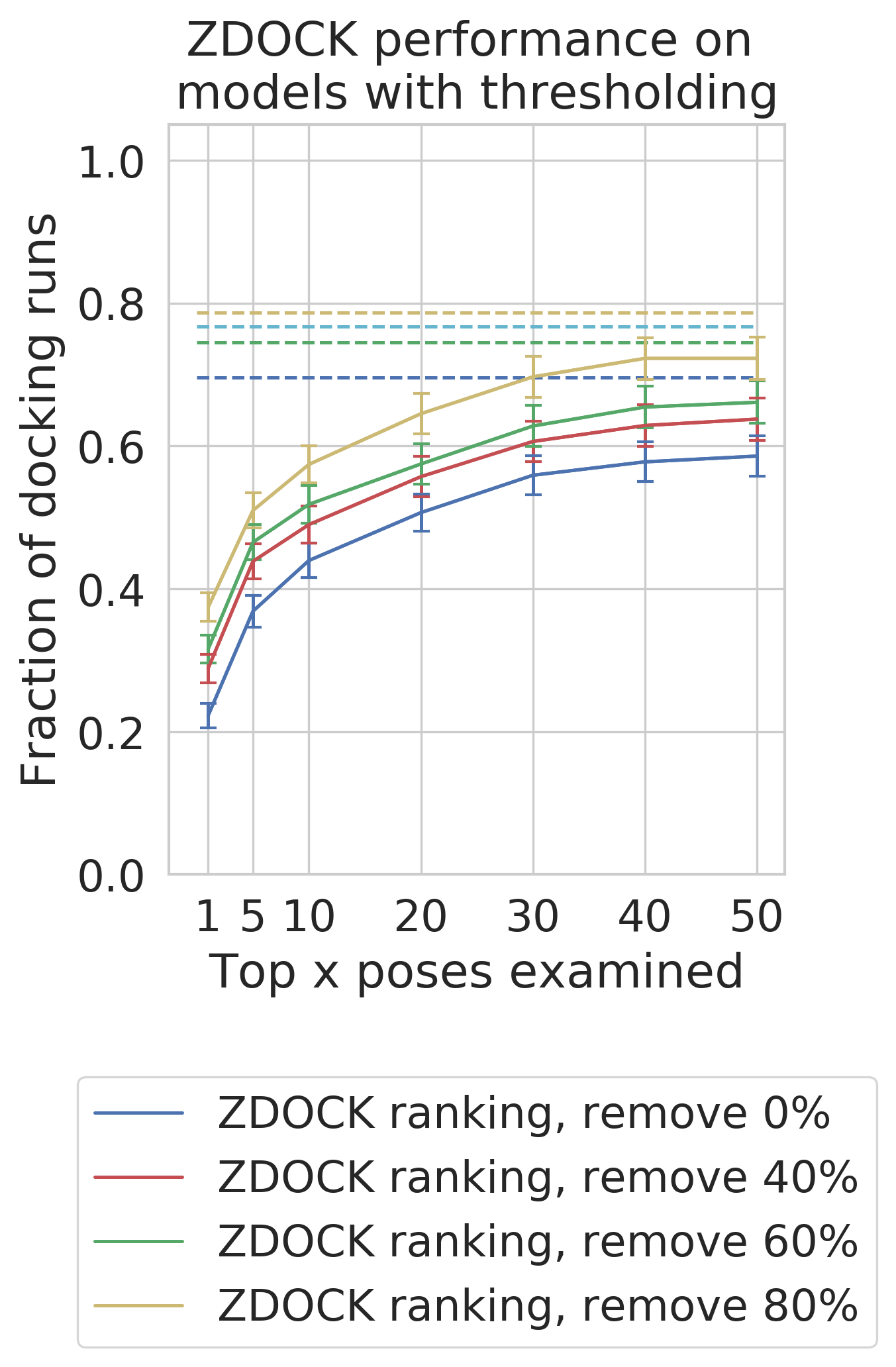

Supplement: btab660_Supplementary_Data [file btab660_supplementary_data.zip › supplementary_figure_8.png]

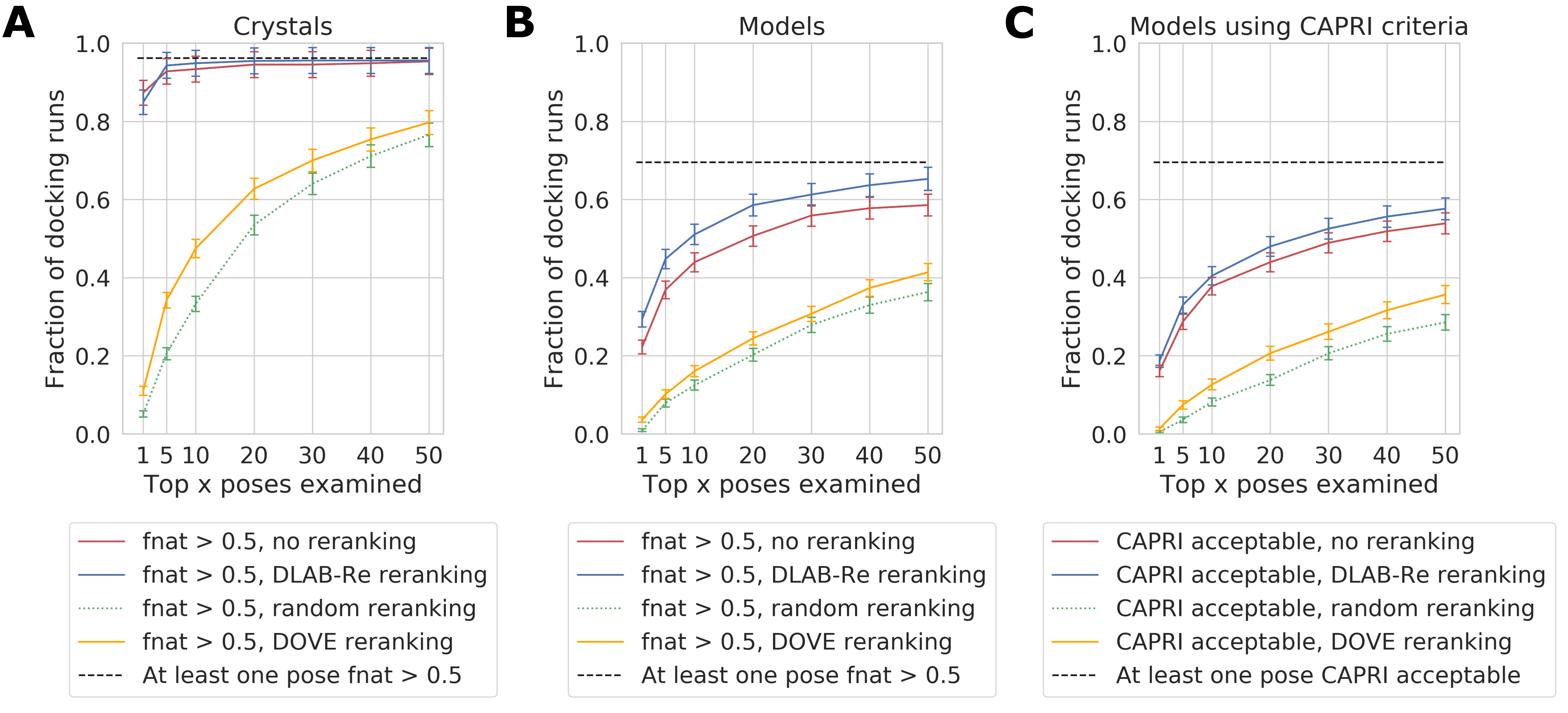

Supplement: btab660_Supplementary_Data [file btab660_supplementary_data.zip › supplementary_figure_9.png]
